# Combinational PRR Agonists in Liposomal Adjuvant Enhances Immunogenicity and Protective Efficacy in a Tuberculosis Subunit Vaccine

**DOI:** 10.3389/fimmu.2020.575504

**Published:** 2020-09-30

**Authors:** Ling Hao, Yaqi Wu, Yandi Zhang, Zijie Zhou, Qing Lei, Nadeem Ullah, Jo-Lewis Banga Ndzouboukou, Xiaosong Lin, Xionglin Fan

**Affiliations:** Department of Pathogen Biology, School of Basic Medicine, Tongji Medical College, Huazhong University of Science and Technology, Wuhan, China

**Keywords:** tuberculosis, subunit vaccine, Bacillus Calmette-Guerin, adjuvant, primary infection, pattern-recognition receptor agonist, monophosphoryl lipid A, trehalose-6,6’-dibehenate

## Abstract

Bacillus Calmette-Guerin (BCG) is the only licensed vaccine to prevent children from tuberculosis (TB), whereas it cannot provide effective protection for adults. Our previous work showed a novel vaccine candidate, liposomal adjuvant DMT emulsified with a multistage antigen CMFO, could protect mice against primary progressive TB, latency, and reactivation. To develop a more effective vaccine against adult TB, we aimed to further understand the role of pattern recognition receptor (PRR) agonists monophosphoryl lipid A (MPLA) and trehalose-6,6’-dibehenate (TDB) of the liposomal adjuvant DMT in the CMFO subunit vaccine-induced protection. Using C57BL/6 mouse models, the current study prepared different dimethyldioctadecylammonium (DDA)–based liposomal adjuvants with MPLA, TDB, or both (DMT), and then compared the immunogenicity and the protective efficacy among different liposomal adjuvanted CMFO subunit vaccines. Our study demonstrated that CMFO/DMT provided stronger and longer-lasting protective efficacy than the CMFO emulsified with adjuvants DDA or DDA/TDB. In addition, DDA/MPLA adjuvanted CMFO conferred a comparable protection in the lung as CMFO/DMT did. Higher levels of IFN-γ, IL-2, TNF-α, and IL-17A secreted by splenocytes were related with a more powerful and durable protection induced by CMFO/DMT through a putative synergistic effect of both MPLA and TDB *via* binding to TLR4 and Mincle. IL-2^+^ CD4^+^ T cells, especially IL-2^+^ CD4^+^ T_CM_ cells, in the lung after infection were significantly associated with the vaccine-induced protection, whereas stronger IL-10 response and lower IL-2^+^ CD4^+^ T cells also contributed to the inferior protection of the DDA/TDB adjuvanted CMFO subunit vaccine. Given their crucial roles in vaccine-induced protection, combinational different PRR agonists in adjuvant formulation represent a promising strategy for the development of next-generation TB vaccine.

## Introduction

Despite the only licensed vaccine to prevent children from tuberculosis (TB), Bacillus Calmette-Guerin (BCG) vaccine cannot generate lifelong immunity, which has a limited protection period of no more than 15 years (1). Currently, adult is a major target population in pulmonary TB epidemics, which accounts for about 90% of the global TB burden (2). Moreover, about one-fourth of the world population has been estimated to be a status of latent TB infection (LTBI) and 5%–10% of them would progress to active TB disease during lifetime (3). Such a situation is currently being exacerbated by the emergence of multidrug-resistant TB (MDR-TB) and extensively drug-resistant TB (XDR-TB), and co-infection with HIV, respectively. As a major threat on global public health, a more effective vaccine is urgently needed to control adult TB.

Attempts have been made to develop novel TB vaccines, such as subunit vaccines, recombinant BCG vaccines, recombinant viral vectors, and attenuated strains, etc. (4). Among them, TB subunit vaccine has attracted increasing attention owing to its definite components and good safety. To produce a robust immune response to reduce the burden of *Mycobacterium tuberculosis* strains under various metabolic states *in vivo*, we and others constructed multistage subunit vaccines, such as A1D4 (Rv1813-Rv2660c-Ag85B-Rv2623-HspX) (5), WH121 (Rv3407-PhoY2-Ag85A-Rv2626c-RpfB) (6), CMFO (Rv2875-Rv3044-Rv2073c-Rv0577) (7), ID93 (Rv3619-Rv1813-Rv3620-Rv2608) (8), and H56 (Ag85B-ESAT-6-Rv2660c) (9), through combining antigens expressed by logarithmically growing and dormant *M. tuberculosis* strains. However, only the antigen CMFO emulsified with the novel liposome adjuvant DMT was validated to be an effective booster of the BCG vaccine (7, 10). Recent clinical trials showed that the efficacy of subunit vaccine candidates M72/AS01_E_ (11) and H4:IC31 (12) to protect against adult TB was only 49.7% and 30.5%, respectively. The imperfect efficacy of clinical trials spurs us on to greater efforts to understand the action mechanism of these candidates.

A significant proportion of adults have already received the BCG vaccination or have been latently infected with *M. tuberculosis* worldwide (3, 13). Under this context, cell-mediated immunity might be more required to play a critical role in the vaccine-induced protection. However, there is still a lack of effective adjuvants to induce appropriate cellular immune responses. The role of adjuvant as a decisive factor affecting the efficacy of TB subunit vaccine is often overlooked. The adjuvant DMT is formulated through the incorporation of dimethyldioctadecylammonium (DDA) liposome by toll-like receptor 4 (TLR4) and Mincle agonists, monophosphoryl lipid A (MPLA) and trehalose-6,6’-dibehenate (TDB) (14–16). The liposomal adjuvant AS01_E_ is composed of MPLA together with QS-21 (a triterpene saponin purified from *Quillaja saponaria*) (11). Another liposome-based adjuvant CAF01 also makes advantage of similar components such as DDA and TDB (17). The common component MPLA, a detoxified version of lipopolysaccharides, can be recognized by pattern recognition receptor (PRR) TLR4 expressing on the surface of antigen-presenting cells (APCs), which activates NF-ĸB through MyD88 and TRIF-dependent pathways and thus induces a Th1 biased response (14, 18–20). The other ingredient TDB, a synthetic analogue of mycobacterial cord factor, binds to the C-type lectin receptors Mincle and Mcl to activate macrophages (21) and could induce MyD88 and Card9-dependent Th1/Th17 responses *in vivo* against *M. tuberculosis* challenge (22, 23). In particular, these adjuvants and their ingredients have been demonstrated to be safe and tolerable in clinical trials (11, 17, 24). We assumed that different PRR agonists might modulate the adjuvant effects of the liposomes and thus affect the efficacy of TB subunit vaccines. To develop a more effective vaccine against TB, we aimed to further understand the role of both PRR agonists of the adjuvant DMT in vaccine-induced protection. In this study, we prepared different DDA-based liposomal adjuvants with MPLA, TDB, or both in this study, and then compared the immunogenicity and the protective efficacy among different liposomal adjuvanted CMFO subunit vaccines in C57BL/6 mouse models.

## Materials and Methods

### Preparation of Liposomal Adjuvants and Vaccines

Four liposomal formulations (**Table S1**), namely, DDA, DDA/MPLA (DM), DDA/TDB (DT), and DMT, were prepared using the lipid film hydration method as previously described (25). Briefly, weighed amounts of DDA (Avanti Polar Lipids Inc., AL, USA), MPLA (Avanti), or TDB (Avanti) were first dissolved in chloroform/methanol (9:1 in volume). The solvent was then blow-dried with N_2_ to form a thin lipid film by using a roto-evaporator. Samples were further dried under hypobaric condition overnight. Unilamellar vesicles were formed by hydrating the lipid film in sterile Tris-buffer (10 mM, pH 7.4) at 60°C for 60 min, followed by vortex every 10 min. Recombinant CMFO protein was expressed by a genetically engineered expression system in *E. coli* and purified using nitrilotriacetic acidmetal ion affinity chromatography (GE Healthcare, NJ, USA) (7). The endotoxin in each purified products was removed (<0.1 EU/ml) by ToxinEraser™ Endotoxin Removal Kit (Genscript, Nanjing, China). Different vaccines were prepared by mixing 100 μl of CMFO solution (0.2 mg/ml) with 100 μl liposomes (**Table S1**). Physicochemical property analysis of both liposomes and vaccine formulations were performed as our previously described (25). The results of the particle size, polydispersity index (PDI), and zeta potential from three batches of samples were presented as mean ± SD.

### Mice and Immunization

Specific-pathogen-free female C57BL/6 mice, 6–8 weeks old, were obtained from the Charles River Company (Beijing, China) and maintained in animal feeding cabinet (VentiRack, CA, USA) in an ABSL-3 biosafety laboratory. Mice were randomly divided into different groups and immunized subcutaneously (s.c.) with different vaccine formulations (200 μl/dose) twice in a 3-week interval. PBS, different liposomal adjuvants DDA, DM, DT, and DMT alone were used as controls. Approximately, 1 × 10^6^ CFU of BCG China strain was vaccinated once as a positive control. All experiments were repeated twice.

### Challenge With Virulent *M. tuberculosis* H37Rv Strain

To evaluate the short-term and long-term protective efficacy, mice vaccinated with different formulations were challenged intranasally (i.n.) with ~100 CFU of virulent *M. tuberculosis* H37Rv strain at the 10^th^ and 20^th^ weeks. Four weeks post-challenge, the protective efficacy among different groups was assessed by comparing bacterial loads in both spleen and lung (n = 6), and by scoring the lung histopathological changes as previously described (n = 3) (5). Briefly, bacterial load per organ was enumerated by plating 10-fold continuous dilutions of whole organ homogenates on 7H11 agar plates (Cat#212203, BD Biosciences, NJ, USA). In addition, 2 µg/ml of 2-thiophenecarboxylic acid hydrazide (Beijing Luqiao Corp, China) was selectively added to inhibit the residual BCG growth. The results were shown as Log_10_ CFU/organ of individual animals (n = 6). The score was obtained by measuring the percentage of the consolidation area of the whole field of vision (magnification ×40) and expressed as mean ± SD of five fields of vision from each group (n = 3).

### Antibody Titer Determination of Antigen-Specific IgG and Its Subclasses

Nine weeks after immunization, CMFO-specific endpoint titers for IgG, IgG1, and IgG2a (Cat#151276, 133045, and 157720; Abcam, Cambridge, MA, USA) were detected in sera from each mouse by ELISA as previously described (7). The results were shown as Log_10_ (endpoint titer) of individual animals (n = 6).

### Determination of Cytokines Secreted by Splenocytes

Nine weeks after immunization or 4 weeks after infection, splenocytes from each mouse were aseptically seeded in triplicate in 24-well plates at the density of 5 × 10^6^ cells/well. The cells were re-stimulated with 10 μg CMFO for 72 h. Culture supernatant was then collected and the cytokines secreted by splenocytes were detected using Mouse Th1 (IFN-γ, IL-2, and TNF-α), Th2 (IL-4), Th17 (IL-17A), regulatory (IL-10 and IL-6) Cytokine Kit (BD Biosciences) based on cytometric bead array (CBA) technology (25).

### Detection of CMFO-Specific T Cells

Nine weeks after immunization or four weeks after infection, intracellular flow cytometry was performed as previously described (7). Briefly, 5 × 10^6^ splenocytes or lung cells from each mouse were seeded in triplicate in 24-well plates and incubated with CMFO (10 μg) and anti-CD28/CD49d (1 μg, eBioscience CA, USA) for 4 h. Then, Brefeldin A (3 μg) and monensin solution (2 μM, eBioscience) were added for further incubation for 12 h. RPMI 1640 medium (Hyclone, USA) was used as a negative control. Cell stimulation cocktail (1 μg, eBioscience) was used to monitor cell responses. Then, cells were collected and stained for 30 min at room temperature in the dark with surface markers, including anti-CD4-APC-Cy7 (Cat#552051, BD Pharmingen™), anti-CD8α-BV510 (Cat#563068, BD Horizon™), anti-CD44-FITC (Cat#561859, BD Pharmingen™), and anti-CD62L-PerCP-Cy5.5 (Cat#560513, BD Pharmingen™). After permeabilization using a Fixation/Permeabilization Solution Kit (Cat#555028, BD Cytofix/Cytoperm™ Plus), cells were stained with intracellular markers, anti-IFN-γ-PE (Cat#554412, BD Pharmingen™) and anti-IL-2-APC (Cat#554429, BD Pharmingen™), for 30 min at room temperature in the dark. Stained cells (5 × 10^5^) were collected and examined by an LSRII multicolor flow cytometry (BD Biosciences). FlowJo software (Tree Star Inc., OH, USA) was used to analyze the proportion of CMFO-specific IFN-γ^+^ (or IL-2^+^) T cells, central memory T cells (T_CM_, CD62L^hi^CD44^hi^), and effector memory T cells (T_EM_, CD62L^lo^CD44^hi^) per organ. The absolute number of each T cell subpopulation was obtained by multiplying its proportion by the total number of the organ cells.

### Statistical Analyses

Statistical analyses were performed using GraphPad Prism 5.0 (San Diego, CA, USA). Two-tailed student’s *t*-test was used for two-group comparison. Multigroup analyses were carried out by one-way ANOVA test, and Tukey’s multiple comparison test was used for further pair-wise comparison. A significant difference was considered when a *p* value was less than 0.05.

## Results

### Physicochemical Characteristics of Both Liposomes and CMFO-Liposome Complexes

**Figure 1:**
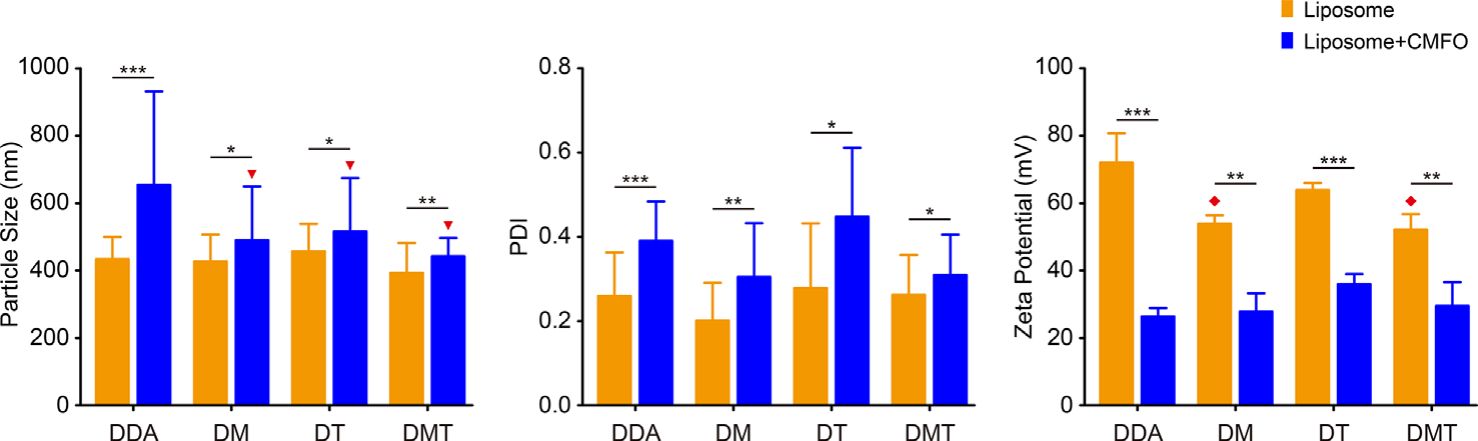
The particle size, PDI, and zeta potential of both liposomes and CMFO-liposome complexes. Results were shown as mean ± SD of three independent liposome batches. ^*^ *p* < 0.05, ^**^ *p* < 0.01, ^***^ *p* < 0.001, ^▾^ *p* < 0.01 vs. CMFO/DDA, and ^♦^ *p* < 0.05 vs. DDA.

Different liposomes had a similar morphology and formed nearly spherical vesicles as our previous demonstrated by transmission electron microscopy (data not shown) (25). Compared with the DDA liposome, an addition of TDB and/or MPLA into the DDA liposome did not result in the change of particle size and PDI (**Figure 1**). In line with previous studies (25, 26), the incorporation of MPLA into DDA vesicles resulted in a significant decrease of the surface charge, as demonstrated by the lower Zeta potential values of DM and DMT. The antigen CMFO, emulsified with different liposomes, resulted in a general trend of increased particle size and PDI while reduced zeta potential across all four formulations. In particular, the particle sizes of CMFO/DM, CMFO/DT, and CMFO/DMT were significantly smaller than that of CMFO/DDA, respectively. However, all CMFO-liposome complexes remained cationic.

### Short- and Long-Term Protection Among Liposomal Adjuvanted Subunit Vaccines

**Figure 2:**
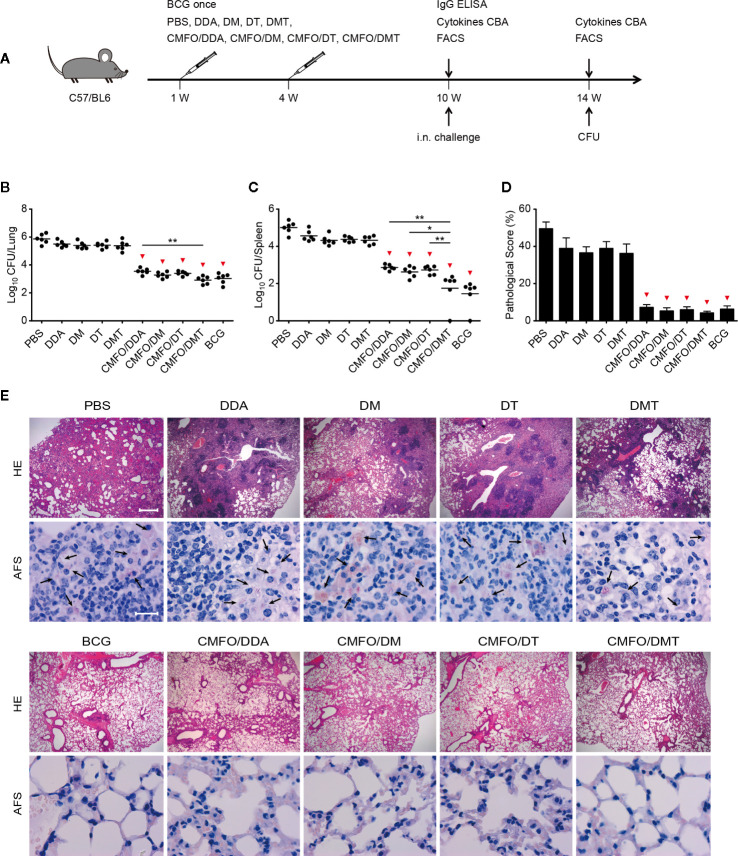
Comparison of the short-term protective efficacy against primary TB infection among different regimens. **(A)** Vaccination and challenge schedule. At the 14^th^ week, bacterial load in the lung **(B)** and the spleen **(C)** of different groups was enumerated and shown as Log_10_ CFU/organ of individual animals (n = 6). The line in each group represented mean value. **(D)** The lung pathological scores of different groups. ^*^ *p* < 0.05, ^**^ *p* < 0.01, and ^▾^ *p* < 0.05 vs. respective controls. **(E)** The representative lung pathological changes were shown for HE and AF staining (n = 3). HE, hematoxylin-eosin; AFS, acid-fast staining. Scar bar: 400 μm for HE staining, 20 μm for AF staining. Arrows indicated AF-positive bacteria. All experiments were repeated twice and similar results were obtained.

To confirm the effect of different adjuvants on the short-term protective efficacy, C57BL/6 mice were vaccinated with different vaccines as described in **Figure 2A** and then challenged with *M. tuberculosis* at the 10th week after immunization. All of liposomal adjuvanted CMFO subunit vaccines resulted in a lower organ bacterial load than their respective adjuvant alone treated controls (**Figures 2B, C** **)**. Notably, CMFO/DMT showed the strongest protection among liposomal adjuvanted CMFO subunit vaccines, as demonstrated by bacterial load in both lung and spleen, lung histopathological changes and scores (**Figures 2B–E** **)**. Consistent with our previous findings (7), there was no statistical difference of bacterial loads in the lung or spleen between CMFO/DMT and BCG groups. Interestingly, when compared with the CMFO/DDA group, CMFO/DMT exhibited a stronger ability to inhibit the growth of *M. tuberculosis* in both lung and spleen, respectively. However, mice vaccinated with CMFO/DMT only had a lower bacterial load in their spleen than CMFO/DM or CMFO/DT did (**Figure 2C**).

**Figure 3:**
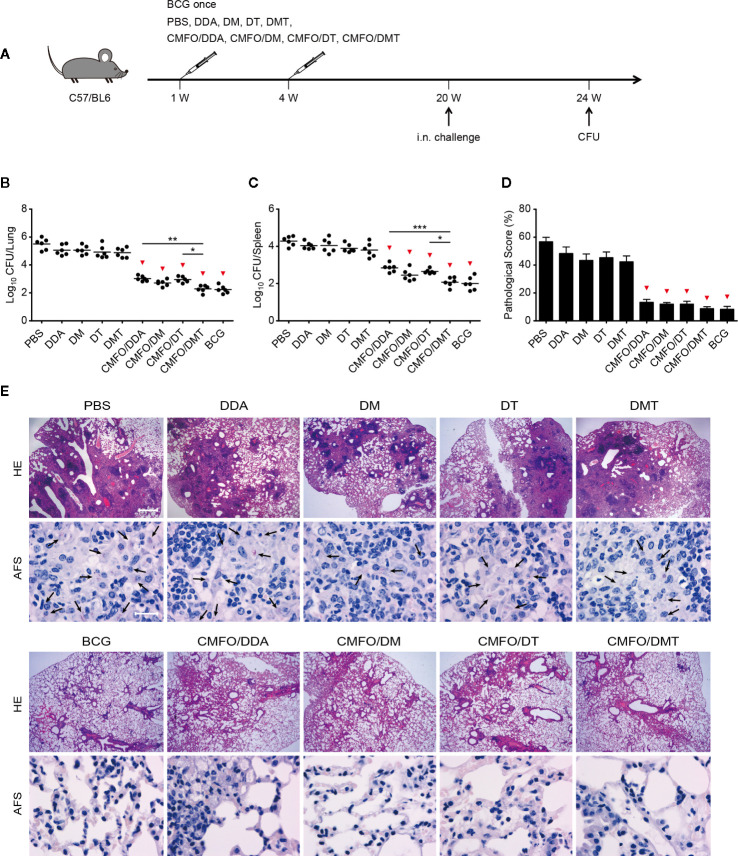
Comparison of the long-term protective efficacy against primary TB infection among different regimens. **(A)** Vaccination and challenge schedule. At the 24^th^ week, bacterial load in the lung **(B)** and the spleen **(C)** of different groups was enumerated and shown as Log_10_ CFU/organ of individual animals (n = 6). The line in each group represented mean value. **(D)** The lung pathological scores of different groups. ^*^ *p* < 0.05, ^**^ *p* < 0.01, ^***^ *p* < 0.001, and ^▾^ *p* < 0.05 vs. respective controls. **(E)** The representative lung pathological changes were shown for HE and AF staining (n = 3). HE, hematoxylin-eosin; AFS, acid-fast staining. Scar bar: 400 μm for HE staining, 20 μm for AF staining. Arrows indicated AF-positive bacteria. All experiments were repeated twice and similar results were obtained.

At the 20^th^ week, vaccinated mice were further challenged with *M. tuberculosis* to examine long-term protective efficacy (**Figure 3A**). Of the all groups, PBS control group still had the highest organ bacterial loads and lung pathological scores. Surprisingly, mice vaccinated with CMFO/DMT had more significantly decreased bacterial load than did with DDA or DT adjuvanted CMFO vaccines (**Figures 3B, C** **)**. In comparison with the CMFO/DM vaccine, CMFO/DMT had milder lung histopathological change and lower score (**Figures 3D, E** **)**. However, both groups had no statistical difference in terms of bacterial load in lung and spleen (**Figures 3B, C** **)**.

### Similar Patterns of Antibody Response Elicited by Liposomal Adjuvanted Subunit Vaccines

**Figure 4:**
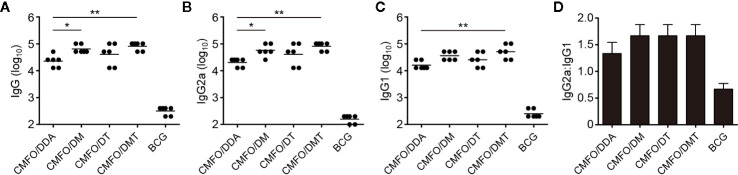
Sera antigen-specific antibody responses (n = 6). Nine weeks after immunization, CMFO-specific endpoint titers for IgG **(A)**, IgG2a **(B),** and IgG1 **(C)** in sera of mice were detected by ELISA. All results were shown as Log_10_ endpoint titer of individual animals and the line in each group represented mean value. ^*^ *p* < 0.05 and ^**^ *p* < 0.01. **(D)** The ratio of IgG2a/IgG1 in different vaccinated mice. All experiments were repeated twice and similar results were obtained.

To analyze the effect of different adjuvants on antibody production, CMFO-specific antibodies, including IgG, IgG2a, and IgG1, in the sera of different vaccinated mice were tested by ELISA. As expected, PBS and adjuvant control groups did not produce any antigen-specific antibodies (data not shown). When compared with the CMFO/DDA group, CMFO/DMT induced much higher levels of CMFO-specific IgG, IgG2a, and IgG1, while CMFO/DM elicited stronger anti-CMFO IgG and IgG2a responses (**Figures 4A–C**). Interestingly, four liposome–based CMFO subunit vaccinated groups induced similar antibody responses, as evidenced by the ratio of IgG2a/IgG1 response to CMFO (**Figure 4D**).

### Differential Cytokine Profiles Among Liposomal Adjuvanted Subunit Vaccines

**Figure 5:**
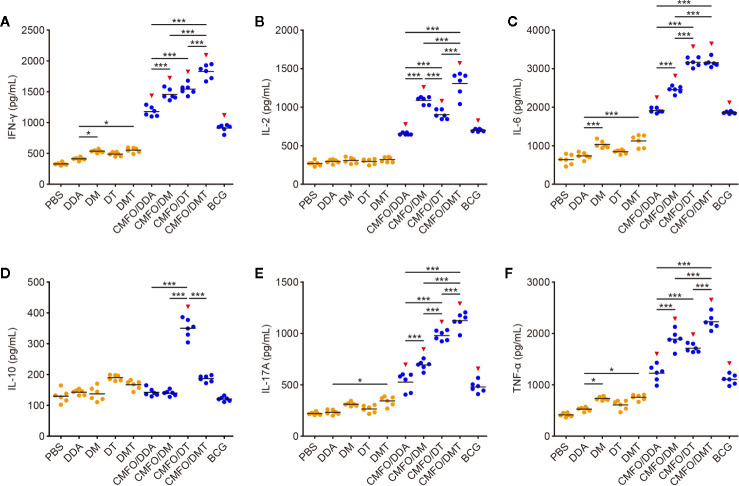
CMFO-specific cytokine responses before exposure (n = 6). Nine weeks after immunization, CMFO-specific Th1/Th2/Th17 cytokines including IFN-γ **(A)**, IL-2 **(B)**, IL-6 **(C)**, IL-10 **(D)**, IL-17 **(E)**, and TNF-α **(F)** in the supernatant of splenocytes from different vaccinated mice were detected by a CBA kit. All experiments were repeated twice and similar results were obtained. The line in each group represented mean value. ^*^ *p* < 0.05, ^***^ *p* < 0.001, and ^▾^ *p* < 0.05 vs. respective controls.

CMFO-specific cytokine profiles in the supernatant of splenocytes from different vaccination groups before and after challenge were detected by using a CBA kit. Prior to the exposure, splenocytes from BCG vaccinated mice secreted the higher levels of CMFO-specific IFN-γ, IL-2, IL-6, IL-17A, and TNF-α than those from the PBS control group (**Figure 5**). When compared with DDA alone, DMT alone significantly increased the levels of CMFO-specific IFN-γ, IL-6, IL-17A, or TNF-α, while DM alone enhanced the secretion of IFN-γ, IL-6, and TNF-α. Different liposomal adjuvanted CMFO vaccinated mice elicited higher levels of IFN-γ, IL-2, IL-6, IL-17A, and TNF-α than their respective adjuvant alone controls. In particular, CMFO/DMT induced the highest levels of IFN-γ, IL-2, IL-17A, and TNF-α of all groups. In addition, mice vaccinated with either CMFO/DM or CMFO/DT also produced more IFN-γ, IL-2, IL-6, TNF-α, and IL-17A than CMFO/DDA did. Only IL-2 response to CMFO in the CMFO/DM group was stronger than that in the CMFO/DT group (**Figure 5B**), while splenocytes from the CMFO/DT vaccinated mice secreted more CMFO-specific IL-6, IL-10, and IL17A than those of the CMFO/DM vaccinated mice (**Figures 5C–E**). Interestingly, CMFO/DT induced the highest level of IL-10 among all liposomal adjvanted CMFO vaccinated mice (**Figure 5D**).

**Figure 6:**
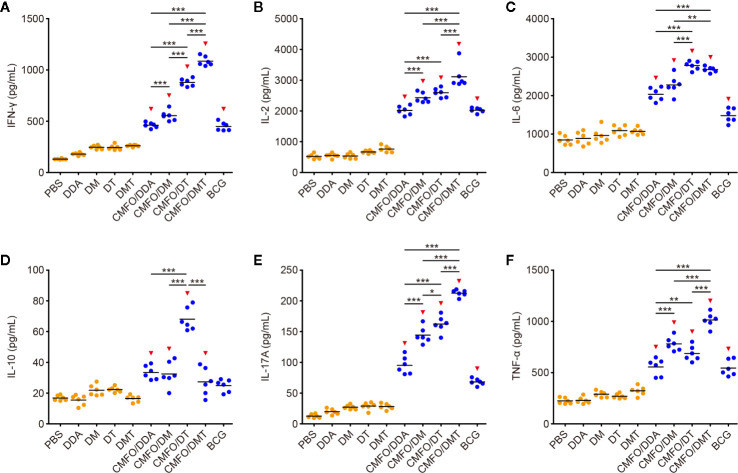
CMFO-specific cytokine responses after exposure (n = 6). Nine weeks after immunization, C57BL/6 mice were challenged with *M. tuberculosis*. Four weeks after infection, CMFO-specific Th1/Th2/Th17 cytokines including IFN-γ **(A)**, IL-2 **(B)**, IL-6 **(C)**, IL-10 **(D)**, IL-17 **(E)**, and TNF-α **(F)** in the supernatant of splenocytes from different vaccinated mice were detected by a CBA kit. All experiments were repeated twice and similar results were obtained. The line in each group represented mean value. ^*^ *p* < 0.05, ^**^ *p* < 0.01, ^***^ *p* < 0.001, and ^▾^ *p* < 0.05 vs. respective controls.

After exposure, the levels of CMFO-specific IL-10 and IL-17A were decreased significantly, whereas IL-2 secretion from splenocytes of different groups had a marked increase (**Figure 6**). Mainly, the results of different groups post-exposure were consistent with those pre-exposure, in addition to the splenocytes from the CMFO/DT vaccinated mice secreted more CMFO-specific IFN-γ, IL-6, IL-10, and IL-17A than those of the CMFO/DM vaccinated mice. Whatever before and after exposure, the level of IL-4 in all groups was very low, less than 1 pg/ml (data not shown).

### Differential T Cell Responses Induced in Spleens Before and After Infection

**Figure 7:**
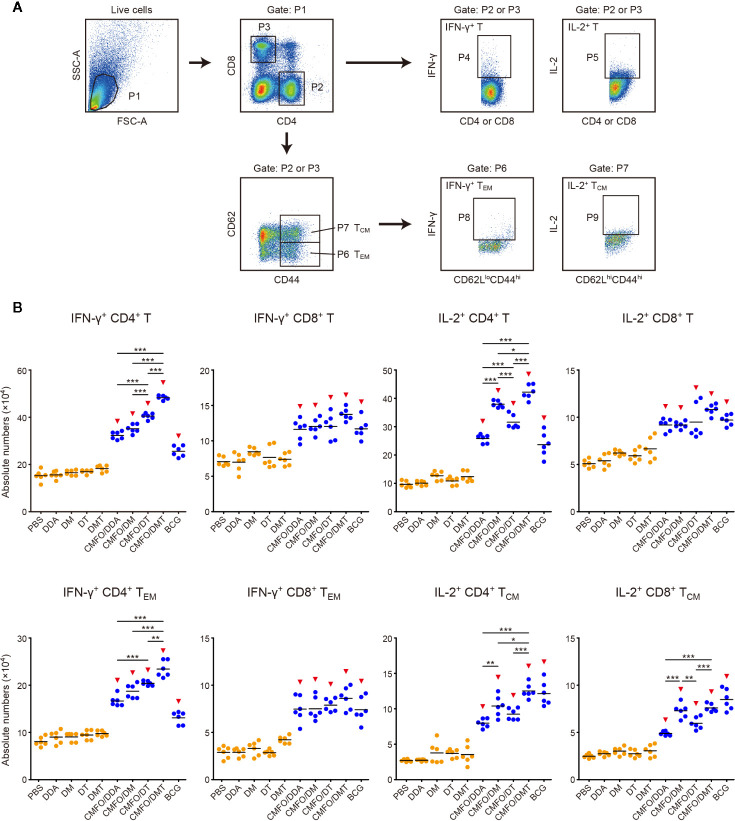
Comparison of the levels of CMFO-specific T cells in the spleen of different immunized mice before exposure (n = 6). Nine weeks after immunization, splenocytes of different groups were collected and stained with different markers for FACS analysis. **(A)** Gating strategy to identify CMFO-specific T cell sub-populations. **(B)** The absolute numbers of CMFO-specific IFN-γ**^+^** CD4^+^ (or CD8^+^) T cells, IL-2^+^ CD4^+^ (or CD8^+^) T cells, IFN-γ^+^ CD4^+^ (or CD8^+^) T_EM_ cells, and IL-2^+^ CD4^+^ (or CD8^+^) T_CM_ cells per spleen of individual animals were shown. All experiments were repeated twice and similar results were obtained. The line in each group represented mean value. ^*^ *p* < 0.05, ^**^ *p* < 0.01, ^***^ *p* < 0.001, and ^▾^ *p* < 0.05 vs. respective controls. Representative FACS plots were shown in **Figure S1**.

**Figure 8:**
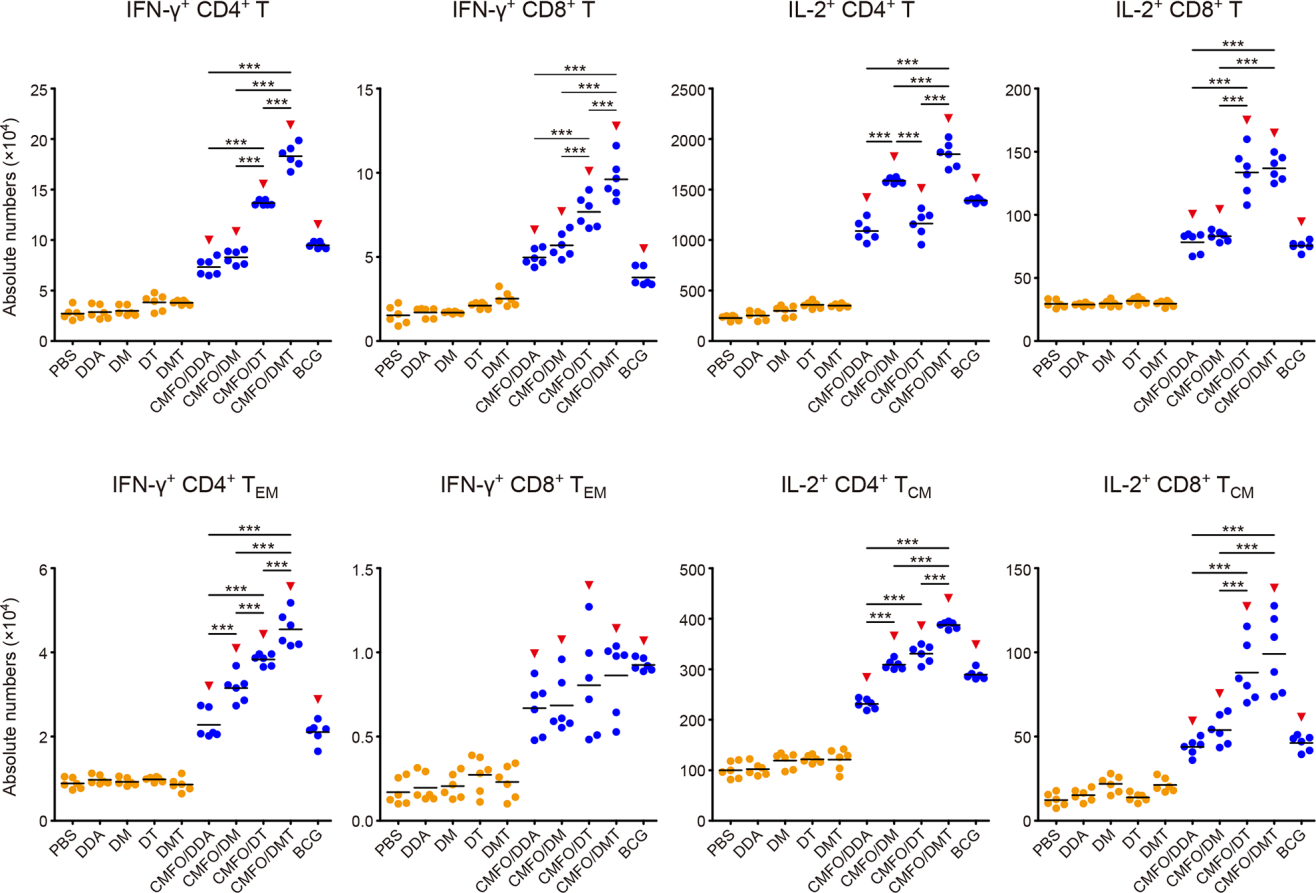
Comparison of the levels of CMFO-specific T cells in the spleen of different immunized mice after exposure (n = 6). Nine weeks after immunization, C57BL/6 mice were challenged with *M. tuberculosis*. Four weeks after infection, the absolute numbers of CMFO-specific IFN-γ**^+^** CD4^+^ (or CD8^+^) T cells, IFN-γ^+^ CD4^+^ (or CD8^+^) T_EM_ cells, IL-2^+^ CD4^+^ (or CD8^+^) T cells, and IL-2^+^ CD4^+^ (or CD8^+^) T_CM_ cells per spleen were detected. The experiments were repeated twice and similar results were obtained. The line in each group represented mean value. ^***^ *p* < 0.001 and ^▾^ *p* < 0.05 vs. respective controls. Representative FACS plots were shown in **Figure S2**.

To investigate immunological effects related with the protection against primary infection, the numbers of CMFO-specific IFN-γ**^+^** (or IL-2^+^) T cells, IL-2^+^ T_CM_ (CD62L^hi^CD44^hi^) cells, and IFN-γ^+^ T_EM_ (CD62L^lo^CD44^hi^) cells in splenocytes from different vaccinated mice were determined by fluorescence activated cell sorting (FACS) before (**Figures 7A, B** **)** and after infection (**Figure 8**). CMFO-specific IFN-γ^+^ CD4^+^ T cells and IFN-γ^+^ CD4^+^ T_EM_ cells were dominated in the spleen of all vaccinated mice before the exposure (**Figure 7B**). As expected, the BCG group had higher numbers of CMFO-specific T cells than that from the PBS control. Liposomal adjuvants alone did not induce any of these T cells at the 10^th^ week. Interestingly, CMFO/DMT induced the highest levels of IFN-γ**^+^** or IL-2^+^ CD4^+^ T cells, IFN-γ^+^ CD4^+^ T_EM_ cells, and IL-2^+^ CD4^+^ T_CM_ cells in the spleen of all groups. When compared with the CMFO/DDA group, CMFO/DM induced more IL-2^+^ CD4^+^ T cells and IL-2^+^ T_CM_ cells, while CMFO/DT induced more IFN-γ**^+^** or IL-2^+^ CD4^+^ T cells and IFN-γ^+^ CD4^+^ T_EM_ cells. More importantly, both DM and DMT adjuvanted CMFO vaccines elicited more IL-2^+^ CD8^+^ T_CM_ cells than CMFO/DDA or CMFO/DT did.

After infection, IL-2^+^ CD4^+^ T cells and T_CM_ cells were dominant in the spleen of all groups (**Figure 8**). Among all groups, CMFO/DMT induced the highest levels of CMFO-specific IFN-γ**^+^** T cells, IFN-γ^+^ CD4^+^ T_EM_ cells, and IL-2^+^ CD4^+^ T cells or T_CM_ cells. When compared with the CMFO/DDA group, CMFO/DM induced more IFN-γ^+^ CD4^+^ T_EM_ cells, IL-2^+^ CD4^+^ T cells, and IL-2^+^ CD4^+^ T_CM_ cells, while CMFO/DT induced more IFN-γ^+^ T cells, IFN-γ^+^ CD4^+^ T_EM_ cells, IL-2^+^ CD8^+^ T cells, and IL-2^+^ T_CM_ cells.

### Differential T Cell Responses Elicited in Lungs After Infection

**Figure 9:**
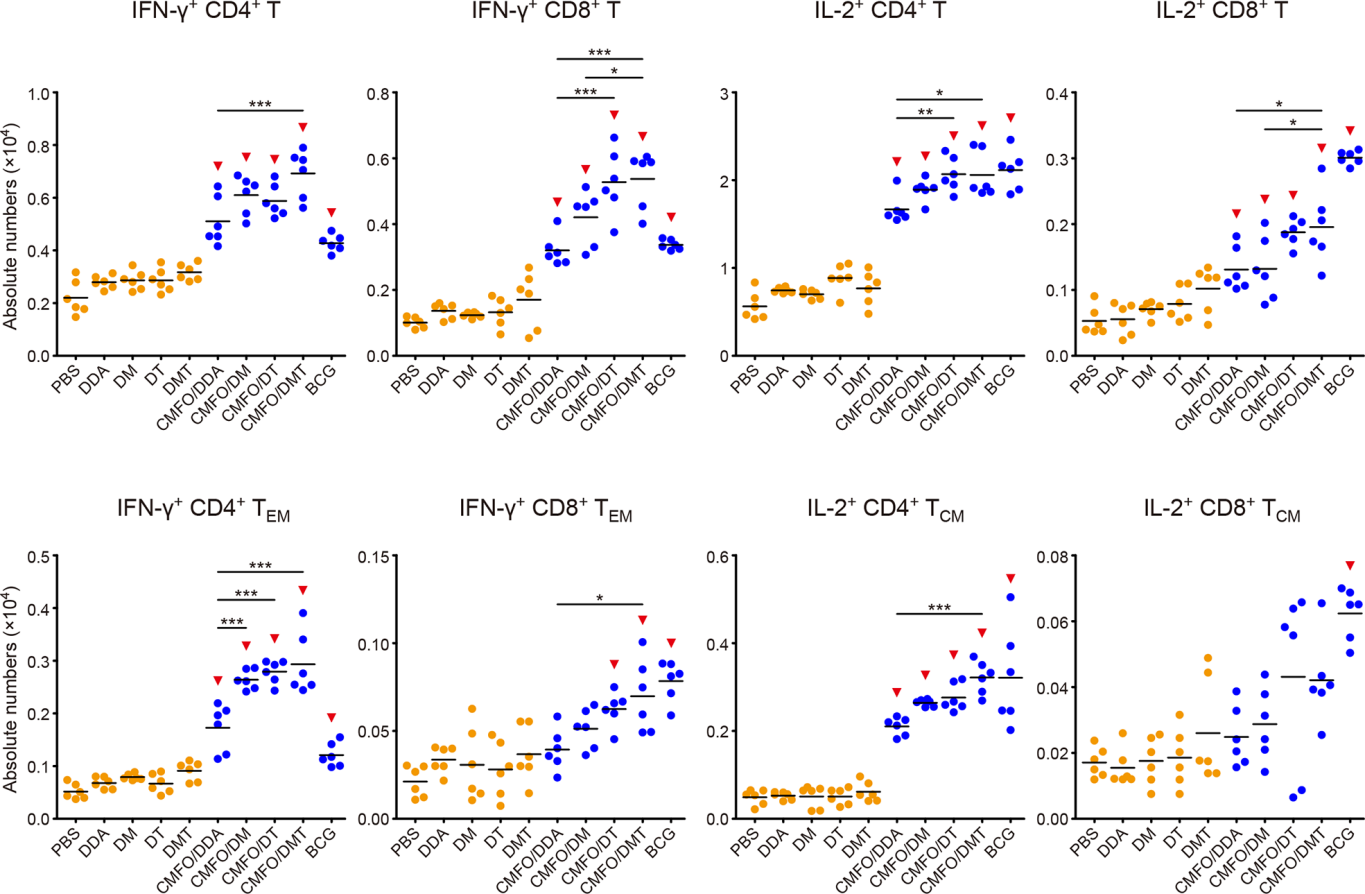
Comparison of the levels of CMFO-specific T cells in the lung of different immunized mice before exposure (n = 6). Nine weeks after immunization, the absolute numbers of CMFO-specific IFN-γ**^+^** CD4^+^ (or CD8^+^) T cells, IFN-γ^+^ CD4^+^ (or CD8^+^) T_EM_ cells, IL-2^+^ CD4^+^ (or CD8^+^) T cells, and IL-2^+^ CD4^+^ (or CD8^+^) T_CM_ cells per lung were detected. The experiments were repeated twice and similar results were obtained. The line in each group represented mean value. ^*^ *p* < 0.05, ^**^ *p* < 0.01, ^***^ *p* < 0.001, and ^▾^ *p* < 0.05 vs. respective controls. Representative FACS plots were shown in **Figure S3**.

**Figure 10:**
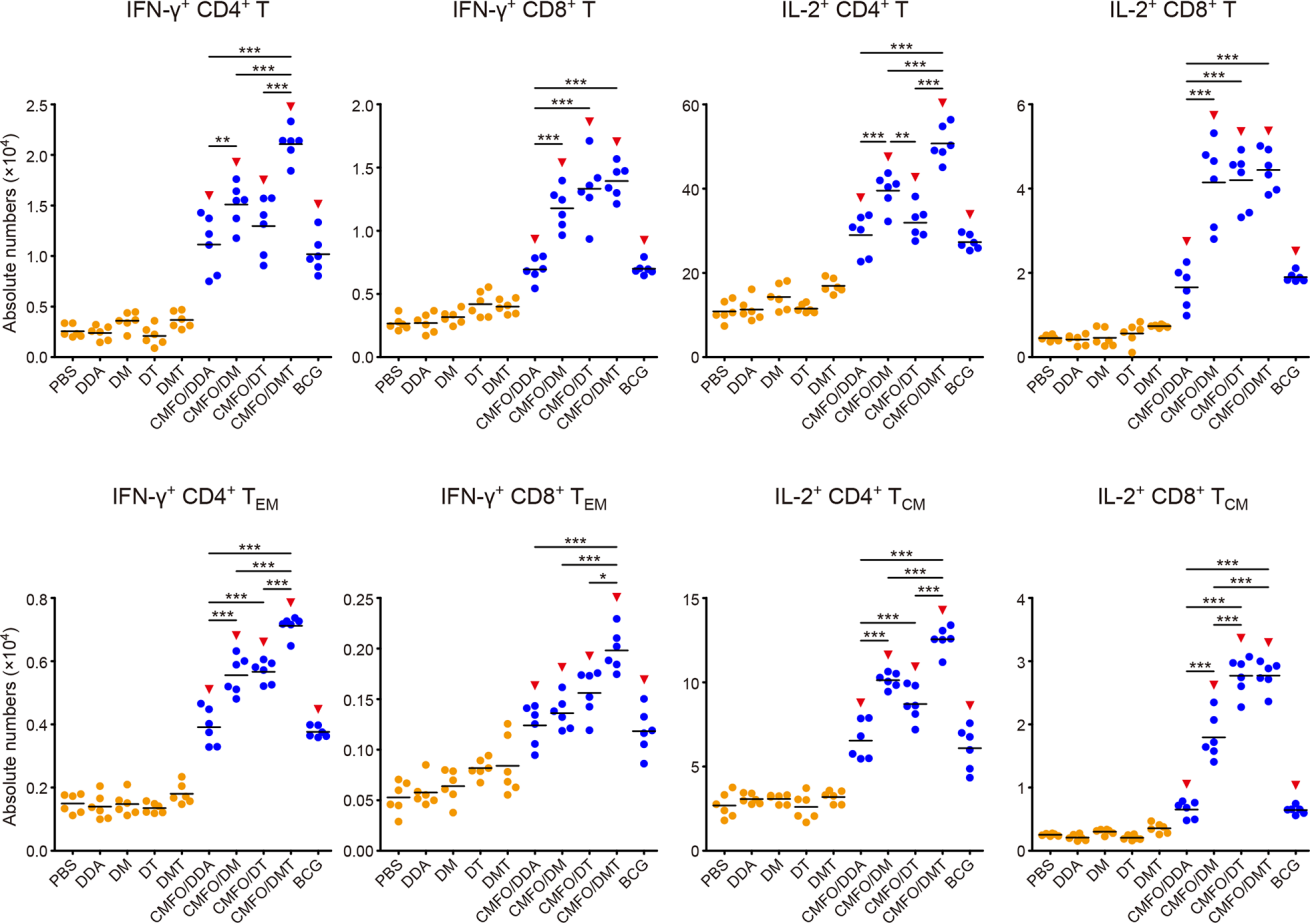
Comparison of the levels of CMFO-specific T cells in the lung of different immunized mice after exposure (n = 6). Nine weeks after immunization, C57BL/6 mice were challenged with *M. tuberculosis*. Four weeks after infection, the absolute numbers of CMFO-specific IFN-γ**^+^** CD4^+^ (or CD8^+^) T cells, IFN-γ^+^ CD4^+^ (or CD8^+^) T_EM_ cells, IL-2^+^ CD4^+^ (or CD8^+^) T cells, and IL-2^+^ CD4^+^ (or CD8^+^) T_CM_ cells per lung were detected. All experiments were repeated twice and similar results were obtained. The line in each group represented mean value. ^*^ *p* < 0.05, ^**^ *p* < 0.01, ^***^ *p* < 0.001, and ^▾^ *p* < 0.05 vs. respective controls. Representative FACS plots were shown in **Figure S4**.

T cell responses to the antigen CMFO were also detected in the lung by FACS (**Figures 9**, **10**). At the 10^th^ week after immunization, IL-2^+^ CD4^+^ T cells were dominated in all vaccinated mice (**Figure 9**). Different adjuvanted CMFO vaccines elicited higher levels of IL-2^+^ CD4^+^ T cells than their respective adjuvant controls. However, the levels of CMFO-specific IFN-γ^+^ T cells or T_EM_ cells, IL-2^+^ CD8^+^ T cells and IL-2^+^ T_CM_ cells in the lung of all groups were very low, only less than 10^4^.

After infection, IL-2^+^ CD4^+^ T cells or T_CM_ cells were still dominated in the lung of all vaccinated groups (**Figure 10**). Notably, CMFO/DMT elicited the highest levels of IFN-γ**^+^** or IL-2^+^ CD4^+^ T cells, IFN-γ^+^ T_EM_ cells, and IL-2^+^ CD4^+^ T_CM_ cells in the lung of all groups. When compared with the CMFO/DDA group, CMFO/DM induced more IFN-γ**^+^** or IL-2^+^ T cells, IFN-γ^+^ CD4^+^ T_EM_ cells, and IL-2^+^ T_CM_ cells, while CMFO/DT induced more IFN-γ**^+^** or IL-2^+^ CD8^+^ T cells, IFN-γ^+^ CD4^+^ T_EM_ cells, and IL-2^+^ T_CM_ cells. In addition, the similar levels of IFN-γ**^+^** or IL-2^+^ CD8^+^ T cells were observed in DM, DT, and DMT adjuvanted CMFO vaccinated groups.

## Discussion

Currently, only a few subunit vaccine candidates with or without prime-boost strategies could exert superior effects than the BCG vaccine does against adult TB in preclinical or clinical trials (27, 28). To develop more effective vaccines, it is significant to understand the role of adjuvants on the efficacy of subunit vaccines. In this study, we investigated the effects of each components of the adjuvant DMT on the protection against primary TB infection in CMFO/DMT subunit vaccinated mice. Our study demonstrated a comparable efficiency between CMFO/DMT and BCG vaccines in terms of their short- and long-term protection. CMFO/DMT achieved a stronger and longer-lasting protection than that from CMFO emulsified with adjuvants DDA or DDA/TDB. Interestingly, DDA/MPLA adjuvanted CMFO could confer to a similar protection in the lung as did with CMFO/DMT. Adjuvants DDA/MPLA, DDA/TDB, and DMT induce similar antibody responses and all are strong inducers of Th1/Th17 cytokine responses. Compared with DMT and DDA/MPLA, the induction of strong IL-10 response and low IL-2^+^ CD4^+^ T cells was relevant to the reduced protection of DDA/TDB adjuvanted CMFO subunit vaccine. Therefore, our findings confirmed that different PRR agonists could modulate the immune responses, especially cellular immune responses in subunit vaccinated mice. The DMT might be a very promising adjuvant for TB subunit vaccines.

**Figure 11:**
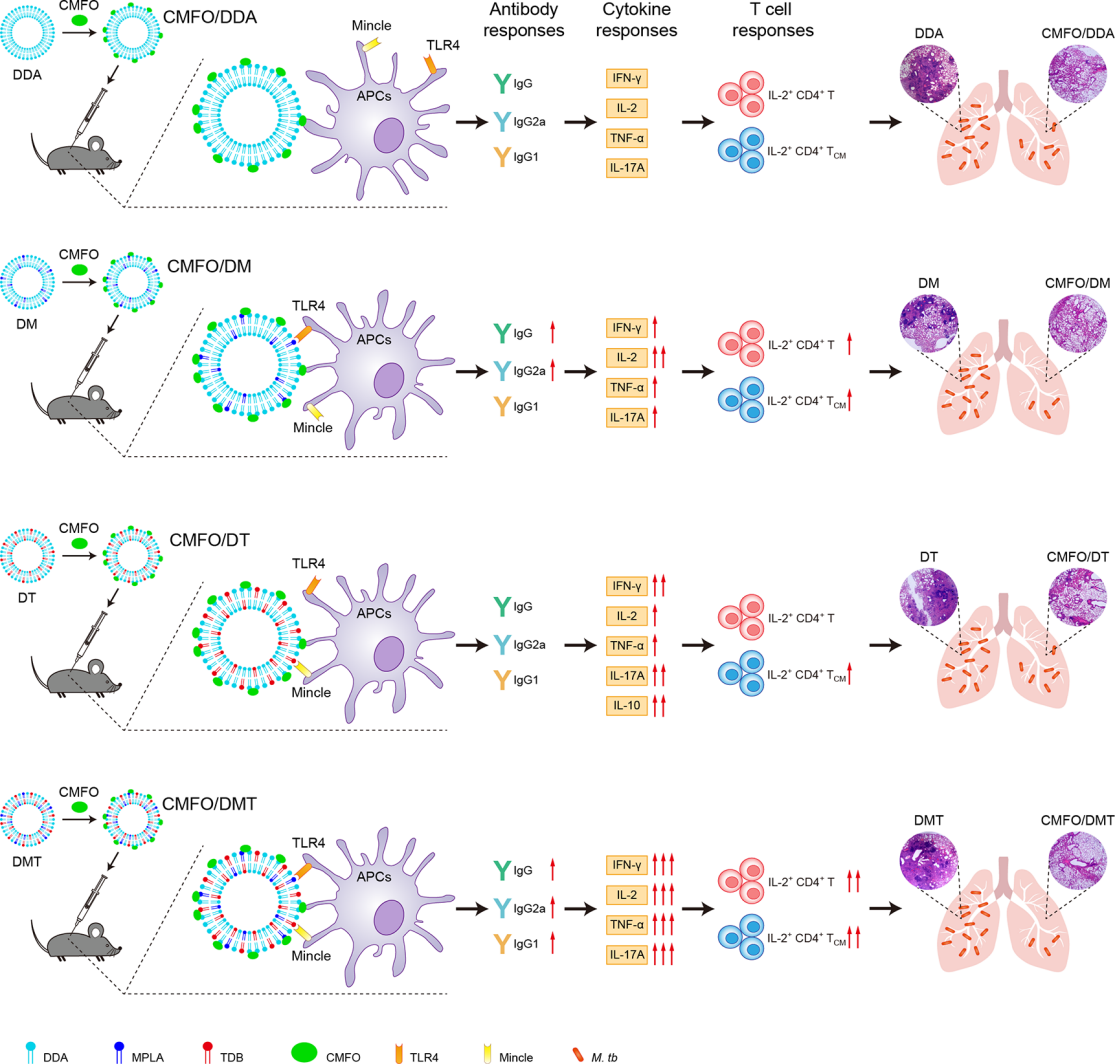
Overview of the mechanism of different liposomal adjuvanted CMFO subunit vaccines.

Differential protective efficacy among liposomal adjuvanted CMFO subunit vaccines provides us opportunities to elucidate immunological mechanisms of different subcomponents in liposomal adjuvants. In this study, the introduction of the negatively charged MPLA into the DDA liposome significantly decreased the surface charge of the liposome, which might improve the stability of DDA-based liposomes as demonstrated in previous studies (25, 26). Cationic adjuvant systems, such as IC31 and CAF01, have been proved to be crucial for the formation of an antigen depot at the site-of-injection, the prolonged uptake of antigens by APCs, and the ability of a vaccine to induce adaptive immune responses (29–31). Our previous study also confirmed that the cationic adjuvant DMT had a slower and longer-lasting release effect on antigens and agonists than the DDA liposome (25). The recombinant antigen CMFO was negative charge, which could be readily adsorbed by the positively charged DMT liposome. The controlled release effect of the DMT adjuvant on antigens and agonists might result in the long-term deposition of the vaccine antigen at the injection site for APCs uptaking, increase the time of vaccine exposure to the immune cells, and thus facilitate the sustained Th1 responses. In the current study, both DM and DMT adjuvanted CMFO subunit vaccines potentiated the production of serum IgG and IgG2b antibodies than the antigen complexed with DDA alone, indicating that antibody-mediated immunity might also play roles in the protection against TB, as previously reported (32–35). The multifaceted functions of the antibody have been proposed as mediating opsonic killing, removing immunomodulatory antigens of *M. tuberculosis* and modulating inflammation (36). Low-antibody titers and defective humoral immunity may increase the risk of *M. tuberculosis* infection and dissemination (37–39). More importantly, different liposomal adjuvanted CMFO vaccines also elicited differential cytokine profiles and T cell responses in the spleen and lung before and after exposure. In line with previous reports (9, 22, 23, 40), the adjuvant DDA/MPLA is a strong inducer of antigen-specific IFN-γ and IL-2 responses, while the adjuvant DDA/TDB stimulated high levels of antigen-specific IFN-γ and IL-17A. However, DDA/TDB also induced the highest level of IL-10 responses to the antigen CMFO before and after infection of all groups. IL-10 suppresses the functions of macrophages and dendritic cells (41, 42), and thus might play a suppressive role in the efficacy of DDA/TDB emulsified CMFO subunit vaccine. Among all groups, DMT adjuvanted CMFO elicited the highest levels of IFN-γ, IL-2, TNF-α, and IL-17A. IFN-γ can trigger the activation of alveolar macrophage, thus killing engulfed *M. tuberculosis* (43–45). TNF-α triggers cytotoxic T cells to directly kill intracellular pathogen, and recruits monocytes and circulate antigen-specific T lymphocytes to the infection site (46, 47). IL-2 is secreted by activated T cells, which can promote the differentiation and proliferation of lymphoid cells, further enhancing the cell-mediated anti-infective immune responses (48). IL-17A plays a critical role in the formation of mature granuloma for pathogen containment at early disease stage (49–51). In addition, IL-6 could induce early IFN-γ expression to inhibit *M. tuberculosis* growth, however it is not necessary for the development of protective immunity (52). Therefore, the CMFO/DMT induced protection correlates with the levels of IFN-γ, IL-2, TNF-α, and IL-17A secreted by splenocytes, which might be a synergistic effect of MPLA and TDB *via* binding to TLR4 and Mincle **(** **Figure 11** **)**. Most importantly, higher levels of effector and central memory T cells correspond to the better vaccine-induced protection against TB as demonstrated in previous studies (7, 17, 53). Differential T cell responses in the spleen and lung before and after exposure were also induced by different subunit vaccines in this study. CD4^+^ T cells play a central role in adaptive immune responses for TB control and even clearance. Vaccine-induced immunological memory is the key to provide lifelong protection. Memory T cells exist in at least two sub-populations, namely, T_EM_ and T_CM_ cells (54, 55). T_EM_ cells express receptors needed for the migration into non-lymphoid organs, which immediately produce microbicidal lymphokines upon reactivation (54, 55). T_CM_ cells express high levels of CCR7, which direct recirculation through lymph nodes and proliferate to produce new antigen-specific CD4^+^ T cells (54, 55). IL-2^+^ CD4^+^ T cells, especially IL-2^+^ CD4^+^ T_CM_ cells might play a pivotal role in vaccine-induced protection as these cells were dominated in the lung of CMFO/DMT vaccinated mice after *M. tuberculosis* infection. In addition, the adjuvants DDA/MPLA and DDA/TDB could elicit different kinds of T cells in the spleen and lung. Depending on the mechanisms of the required protective immunity, these adjuvants can be utilized to develop subunit vaccines for preventing against different infectious diseases.

Taken together, our findings have illustrated a synergistic effect among subcomponents MPLA and TDB of the adjuvant DMT, which together contribute an enhanced immunogenicity and better longer-lasting protection of the CMFO/DMT vaccine against primary progressive TB. Therefore, the current work is an important extension of the CMFO/DMT vaccine. Given a crucial role of adjuvants in vaccine-induced protection, a combinational strategy with different PRR agonists might be a direction deserved for further investigation toward a next-generation TB vaccine.

## Data Availability Statement

The raw data supporting the conclusions of this article will be made available by the authors, without undue reservation.

## Ethics Statement

The animal study was reviewed and approved by the Committee on the Ethics of Animal Experiments and the School Committee on Biosafety of Tongji Medical College, Huazhong University of Science and Technology.

## Author Contributions

This project was designed by XF. LH, YW, ZZ, QL, YZ, NU, J-LBN, and XL performed the experiments. LH, XF, YW, ZZ, and QL analyzed the data. LH wrote the manuscript and the final manuscript was revised thoroughly by XF.

## Funding

This work was supported by grants from the National Mega-Projects of Science Research for the 13th Five-year Plan of China (No. 2018ZX10302302002-001), the Natural Science Foundation of China (No. 81772147, 81971909), the Fundamental Research Funds for the Central Universities (HUST COVID-19 Rapid Response Call No. 2020kfyXGYJ040), and the R&D program of Wuhan Bureau of Science and Technology (No. 2020020601012218).

## Conflict of Interest

The authors declare that the research was conducted in the absence of any commercial or financial relationships that could be construed as a potential conflict of interest.

## Abbreviations

TB, tuberculosis; BCG, Bacillus Calmette-Guerin; DDA, dimethyldioctadecylammonium; MPLA, monophosphoryl lipid A; TDB, trehalose-6,6’-dibehenate; DM, DDA/MPLA; DT, DDA/TDB; DMT, DDA/MPLA/TDB; LTBI, latent TB infection; MDR-TB, multidrug resistant TB; XDR-TB, extensively drug resistant TB; A1D4, Rv1813-Rv2660c-Ag85B-Rv2623-HspX; WH121, Rv3407-PhoY2-Ag85A-Rv2626c-RpfB; CMFO, Rv2875-Rv3044-Rv2073c-Rv0577; ID93, Rv3619-Rv1813-Rv3620-Rv2608; H56, Ag85B-ESAT-6-Rv2660c; PRR, pattern-recognition receptor; TLR4, toll-like receptor 4; antigen-presenting cells, APCs; PDI, polydispersity index; s.c., subcutaneously; i.n., intranasally; CBA, cytometric bead array; FACS, fluorescence activated cell sorting; T_CM_, central memory T cell; T_EM_, effector memory T cell.
