# Supplementary material for: Combinational PRR Agonists in Liposomal Adjuvant Enhances Immunogenicity and Protective Efficacy in a Tuberculosis Subunit Vaccine
Source: Front Immunol. 2020 Sep 30;11:575504. doi: 10.3389/fimmu.2020.575504 (PMC7561437; doi:10.3389/fimmu.2020.575504)
Supplement: Supplementary file 1 [file DataSheet_1.docx]

**Supplementary Materials**

**
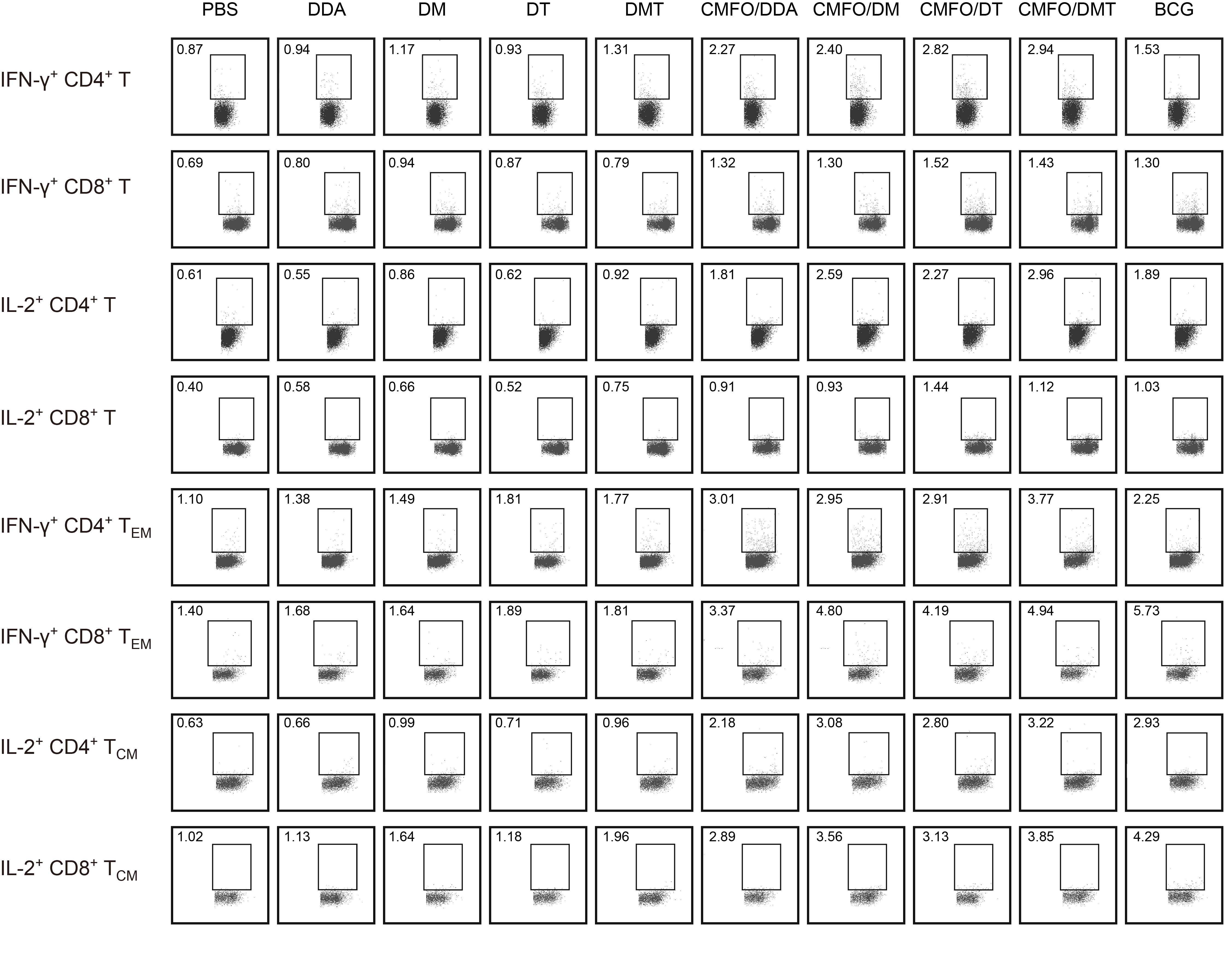
**

**FIGURE S1.** Representative FACS plots of CMFO-specific T cells in the spleen of different immunized mice before exposure.


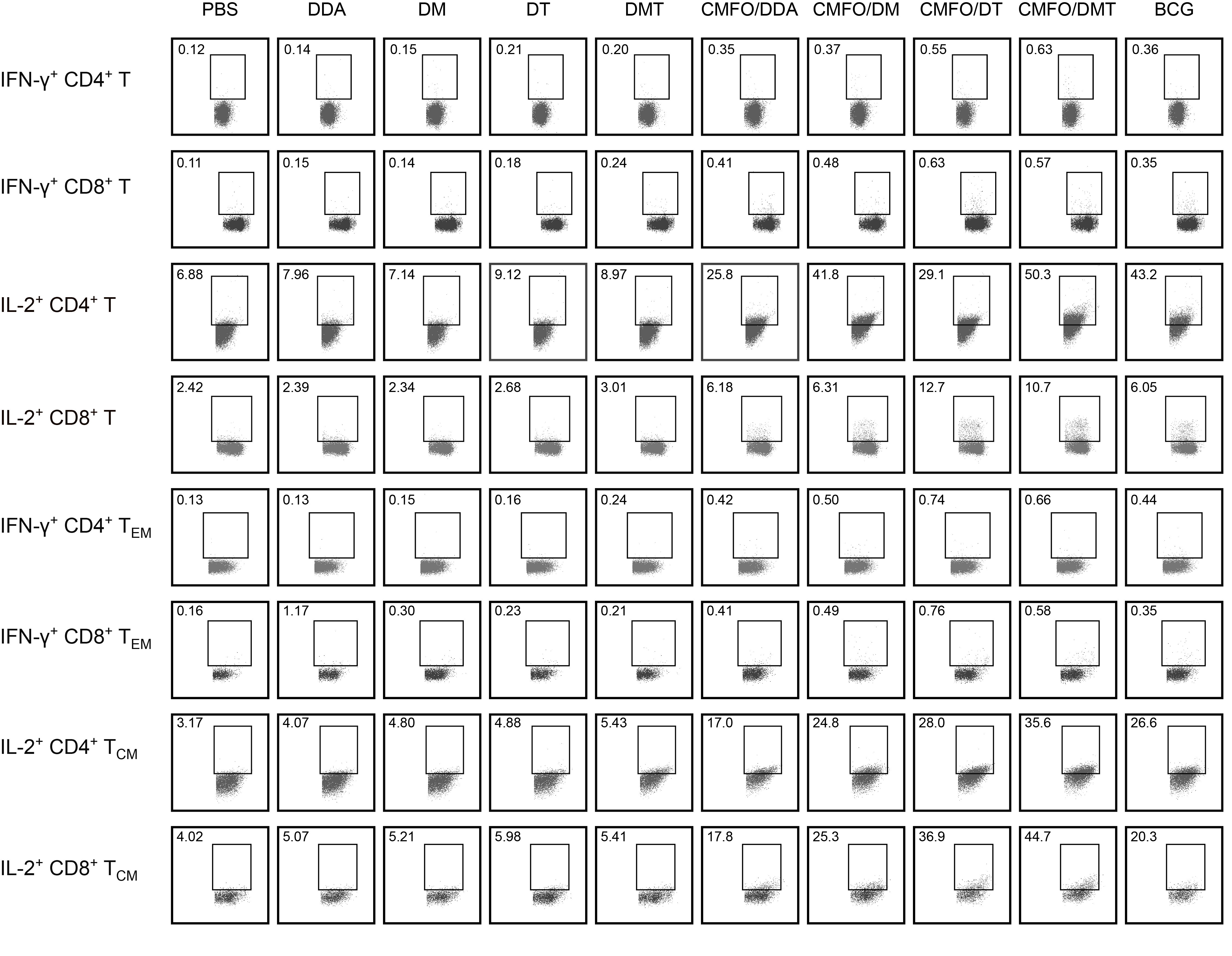


**FIGURE S2.** Representative FACS plots of CMFO-specific T cells in the spleen of different immunized mice after exposure.


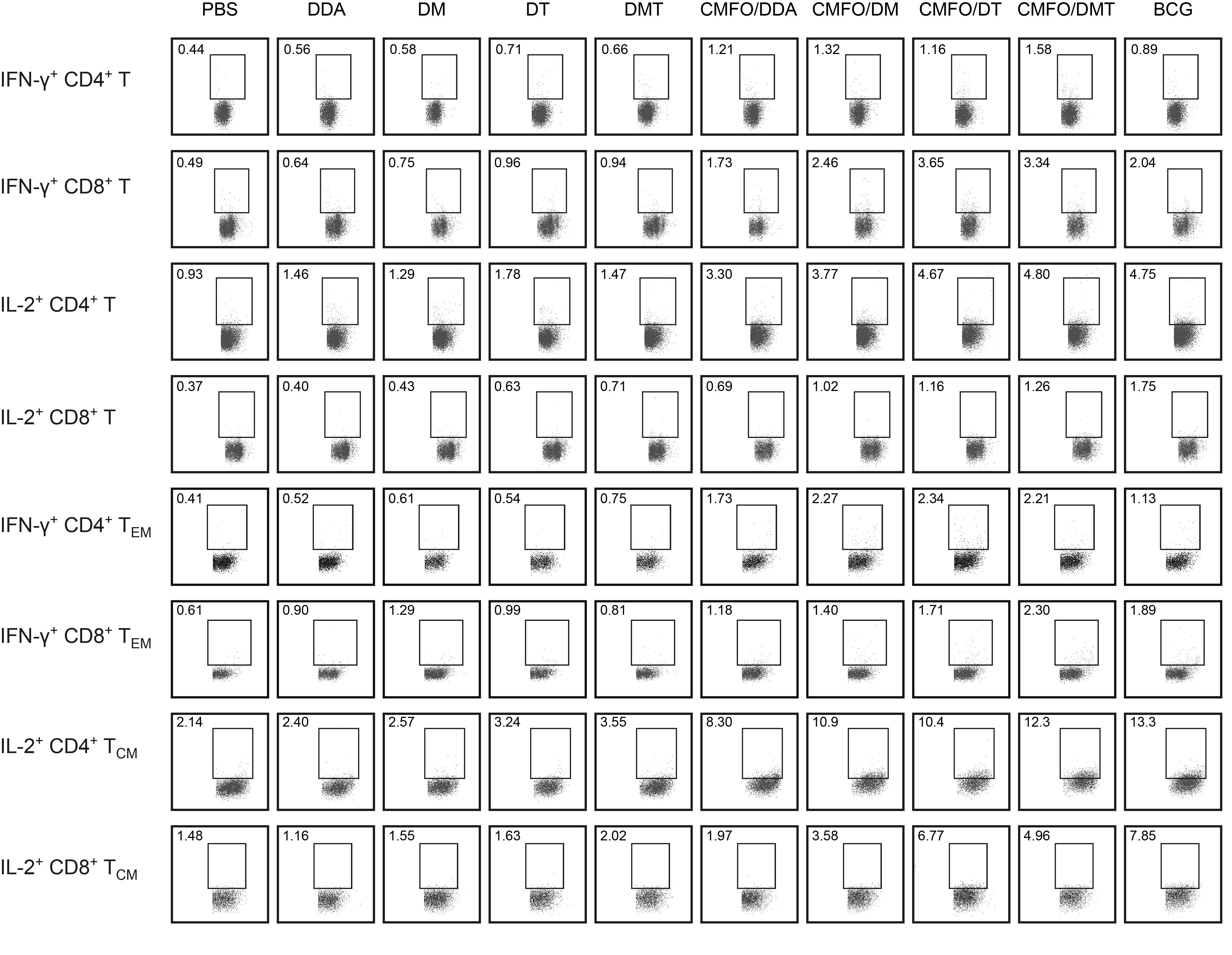


**FIGURE S3.** Representative FACS plots of CMFO-specific T cells in the lung of different immunized mice before exposure.


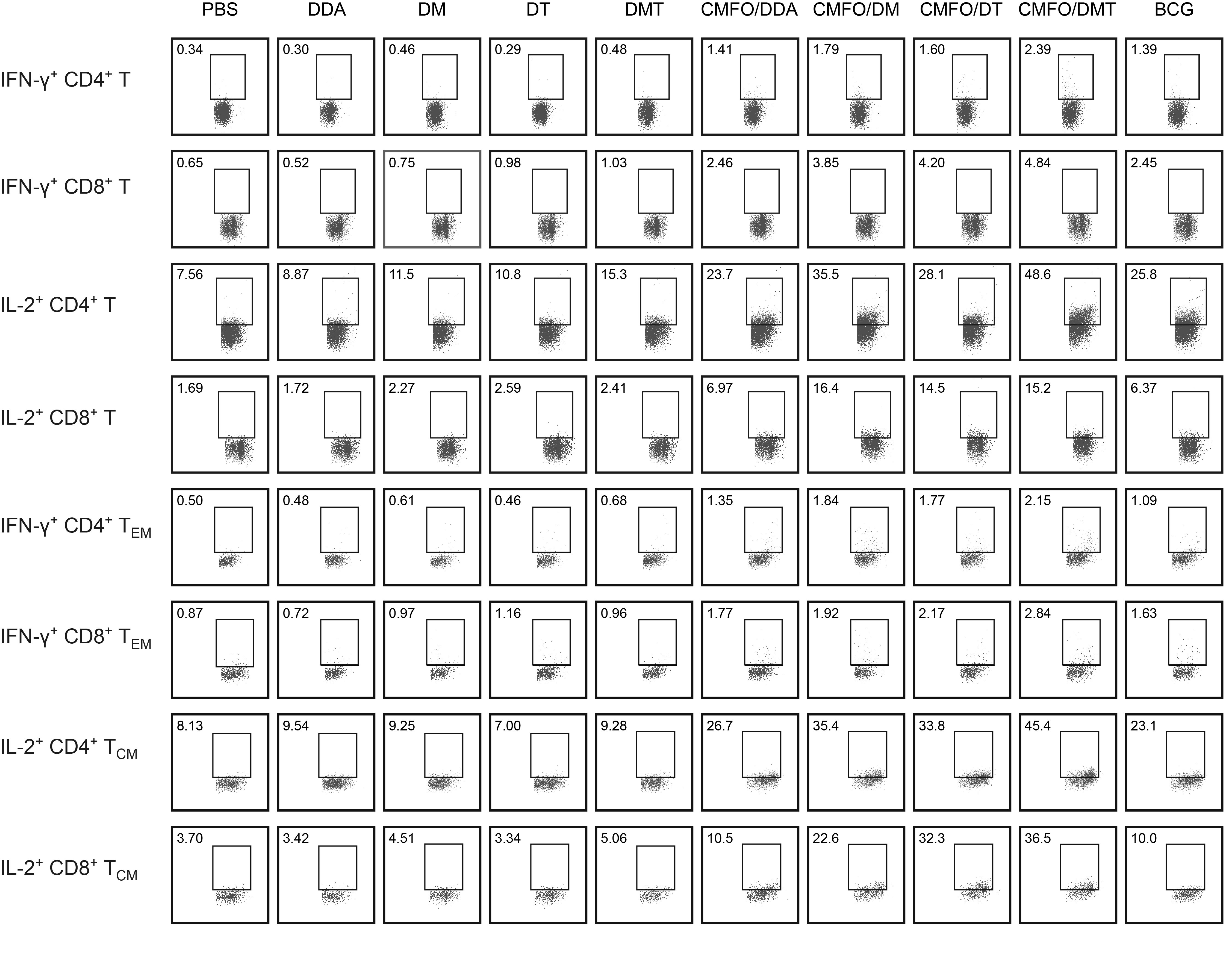


**FIGURE S4.** Representative FACS plots of CMFO-specific T cells in the lung of different immunized mice after exposure.

**Table S1.** Formulations of different liposomal adjuvants and subunit vaccines.

| Name | Composition | Content (mL^-1^) |
| --- | --- | --- |
| DDA | DDA | DDA 2.5 mg |
| DDA/MPLA (DM) | DDA, MPLA | DDA 2.5 mg, MPLA 0.25 mg |
| DDA/TDB (DT) | DDA, TDB | DDA 2.5 mg, TDB 0.5 mg |
| DMT | DDA, MPLA, TDB | DDA 2.5 mg, MPLA 0.25 mg, TDB 0.5 mg |
| CMFO/DDA | CMFO, DDA | CMFO 0.1 mg, DDA 1.25 mg |
| CMFO/DM | CMFO, DDA, MPLA | CMFO 0.1 mg, DDA 1.25 mg, MPLA 0.125 mg |
| CMFO/DT | CMFO, DDA, TDB | CMFO 0.1 mg, DDA 1.25 mg, TDB 0.25 mg |
| CMFO/DMT | CMFO, DDA, MPLA, TDB | CMFO 0.1 mg, DDA 1.25 mg, MPLA 0.125 mg, TDB 0.25 mg |
